# Non-invasive assessment of sublingual microcirculation using flow derived from green light PPG: evaluation and reference values

**DOI:** 10.1117/1.JBO.29.1.017001

**Published:** 2024-01-05

**Authors:** Rafael Uribe Acevedo, Laura Osorio Sánchez, Sebastián Vergara Londoño, Elisa Mejía-Mejía, Róbinson Torres Villa, Yesid Montoya Goez

**Affiliations:** a Universidad EIA, Medellín, Colombia; b Hospital Alma Máter de Antioquia, Servicio de Medicina Crítica y Cuidados Intensivos, Medellín, Colombia; c King’s College London, Centre for Human and Applied Physiological Sciences, London, United Kingdom

**Keywords:** sublingual area, microcirculation, photoplethysmography

## Abstract

**Significance:**

The study of sublingual microcirculation offers valuable insights into vascular changes and overcomes some limitations of peripheral microcirculation assessment. Videomicroscopy and pulse oximetry have been used to assess microcirculation, providing insights into organ perfusion beyond macrohemodynamics parameters. However, both techniques have important limitations that preclude their use in clinical practice.

**Aim:**

To address this, we propose a non-invasive approach using photoplethysmography (PPG) to assess microcirculation.

**Approach:**

Two experiments were performed on different samples of 31 subjects. First, multi-wavelength, finger PPG signals were compared before and while applying pressure on the sensor to determine if PPG signals could detect changes in peripheral microcirculation. For the second experiment, PPG signals were acquired from the ventral region of the tongue, aiming to assess the microcirculation through features calculated from the PPG signal and its first derivative.

**Results:**

In experiment 1, 13 out of 15 features extracted from green PPG signals showed significant differences (p<0.05) before and while pressure was applied to the sensor, suggesting that green light could detect flow distortion in superficial capillaries. In experiment 2, 15 features showed potential application of PPG signal for sublingual microcirculation assessment.

**Conclusions:**

The PPG signal and its first derivative have the potential to effectively assess microcirculation when measured from the fingertip and the tongue. The assessment of sublingual microcirculation was done through the extraction of 15 features from the green PPG signal and its first derivative. Future studies are needed to standardize and gain a deeper understanding of the evaluated features.

## 1 Introduction

The term microcirculation refers to the group of blood vessels that have a diameter less than 100  μm, which includes arterioles, meta-arterioles, capillaries, and venules.^1^^,^^2^ It plays an essential role in delivering oxygen to cells and regulating blood pressure by modifying vascular resistance.^1^^–^^4^ Under normal physiological conditions, the microcirculatory status aligns with macrohemodynamic variables, such as blood pressure, heart rate, and cardiac output. However, in certain pathological states, such as sepsis or during shock resuscitation, this correlation can be disrupted, leading to a condition known as loss of hemodynamic coherence. In this state, microcirculation may be compromised even when macrohemodynamic variables remain within normal limits. This phenomenon is also referred to as microcirculatory shock.^3^^,^^5^^–^^7^

To date, the monitoring of tissue perfusion through the evaluation of peripheral microcirculation has been carried out by employing various methods, among which the following stand out: nail fold capillaroscopy, capillary refill time in the fingertips, laser Doppler flowmetry of the skin, tissue oxygen saturation (${\mathrm{StO}}_{2}$) at the thenar eminence through the resaturation rate ($R_{\mathrm{res}}$) using near-infrared spectroscopy (NIRS), and sublingual videomicroscopy (evaluated in the lateral side of the tongue and the floor of the mouth).^3^^,^^8^^–^^13^

Although NIRS allows the microcirculation assessment by measuring changes in the concentration of tissue oxygenated and deoxygenated hemoglobin (Hb), it requires a vascular occlusion test (VOT), which results in discontinuous measurements, in addition to the inability of reproducing this technique in other body sites that reflects visceral microcirculation state as sublingual area.^13^^–^^15^ On the other hand, the inherent barriers in the peripheral assessment of microcirculation from the finger, such as nail polish, skin color, and the influence of temperature on distal perfusion, along with the crucial relationship between visceral and sublingual microcirculation, have increased the interest in measuring and studying the latter.^8^^,^^16^^,^^17^ There is growing evidence suggesting a link between sublingual microcirculation and vascular changes in shock and sepsis.^9^ Most of these results have been possible due to the introduction and evolution of videomicroscopy techniques, such as orthogonal polarization spectral imaging, sidestream dark field imaging, and incident dark field imaging, which have not only provided clearer visualization of capillaries but also improved image resolution, enabling a more precise characterization of microcirculatory changes, during various physiopathological processes.^12^^,^^18^^–^^23^ It has been suggested that these changes in microcirculation may manifest in early states of sepsis, even before observable alterations in macrohemodynamic variables occur.^5^ Thus, videomicroscopy offers valuable insights into sublingual microcirculation for assessing hemodynamic status and tissue perfusion.^5^^,^^20^^,^^24^ Nonetheless, this technology has some limitations, including the need for specialized equipment and training, the requirement of a proper stabilization of the lens for optimal high-quality images, regular equipment maintenance and time consumption while acquiring and analyzing the microcirculatory images, which can be costly to acquire and maintain, particularly in resource-limited settings, restraining its widespread access in clinical facilities located in low- and middle-income countries.^1^^–^^3^^,^^9^^,^^21^^,^^25^

Photoplethysmography (PPG) based techniques have been employed for the indirect assessment of peripheral microcirculation, typically through oxygen saturation measurements in the thenar eminence.^1^^,^^11^ This approach provides a simpler and more cost-effective option compared to videomicroscopy. However, it is important to recognize that peripheral assessment comes with its own limitations, especially when the resulting value is an estimation rather than a direct measure.^26^^–^^29^ This study presents a non-invasive tool for assessing microcirculation, applying an optical method based on PPG, but not solely based on the measurement of oxygen saturation. The proposed approach involves applying this technique within the oral cavity, thereby retaining the benefits of sublingual region evaluation. In addition to these advantages, the application of PPG-based technology offers ease of use and cost-effectiveness, making it a promising alternative for microcirculation assessment.

## 2 Materials and Methods

### 2.1 Sample Description

Two experiments were performed, with independent samples. For the first experiment, a random sample recruitment approach was implemented in a population of university members over 18 years old, without considering any other eligibility criteria. The study was reviewed and approved by the EIA University’s ethics committee. Signal acquisition was performed in a sample of 31 subjects, after each subject gave written informed consent.

The second experiment was conducted in another group of 31 healthy participants. Subjects were selected based on the predefined eligibility criteria detailed in Table 1. This choice was made to mitigate the impact of pre-existing health conditions or any potential biases, ensuring that the outcomes were not influenced by these factors. These criteria were specifically designed to exclude individuals with pre-existing oral cavity microcirculatory abnormalities resulting from medical conditions, prior surgical procedures, or the use of pharmacologically active substances. Table 2 described the demographic and clinical characteristics of this sample.

**Table 1:** Eligibility criteria for the experiment 2.

**Inclusion**
1. Individuals aged 18 to 55 years old
2. Clinically healthy individuals or those with a stable underlying condition that does not impact the cardiovascular system
3. Participants with a body mass index (BMI) between 18.5 and 29.9
**Exclusion**
• Anatomical deformities in the upper limbs, tongue, and oral cavity
• History of oral surgery
• Intake of dark or energy drinks within 6 h before signal acquisition
• History of hypertension, diabetes, any degree of kidney disease, ischemic heart disease, autoimmune, and rheumatic diseases
• History of smoking or use of psychoactive substances
• Presence of febrile syndrome within the last 72 h
• Any current or recent (within the past 7 days) infectious process
• Current periodontal disease or oral mucosa disease
• Users of dental prostheses or orthodontic devices
• Current use of bronchodilators (B2 agonists, anticholinergics)
• Known pregnancy or absence of menstruation for more than 5 weeks without contraceptive use

**Table 2:** Demographic and clinical characteristics of the subjects on experiment 2.

Characteristic	All patients (n=31)
Age (years) mean ± SD	21.8 ± 4.8
Male sex – no. (%)	13 (41.93)
Female sex – no. (%)	18 (58.06)
Body mass index mean ± SD	23.9 ± 3.4
Systolic blood pressure mean ± SD	106.1 ± 11.3
Diastolic blood pressure mean ± SD	68.2 ± 7.2
Heart rate mean ± SD	81.8 ± 9

### 2.2 Signal Acquisition

In both experiments, a multi-wavelength PPG sensor (MAX30105 Analog Devices Inc./Maxim Integrated) was used to monitor blood volume changes in vessels from the microcirculatory system, using the built-in red, infrared, and green light-emitting diodes (LEDs). The sensor features a sampling rate of 66 Hz and ADC resolution of 16 bits. Given that LEDs with shorter wavelengths have less penetration depth in tissue due to their higher absorption by Hb, there is distinct advantage to LEDs with shorter wavelengths when assessing peripheral microcirculation, such as blue and green wavelengths.^30^ These wavelengths can reach the papillary dermis and vascular structures as blood vessels of the upper dermis.^31^^–^^37^ On the contrary, LEDs with longer wavelengths can reach subdermal layers, which may interfere with the measurement of microcirculation.^31^^–^^37^ Considering this property of light–tissue interaction, the green PPG was acquired and analyzed as part of the evaluation of microcirculation in both experiments.

In the first experiment, PPG signals were acquired from the fingertip. Subjects were seated and instructed to keep their left arm positioned in a supine manner on a desk, with the sensor placed on the fourth fingertip of the same arm. This experiment took place in a dark room to avoid light influence on the acquired signals.

**Fig. 1:**
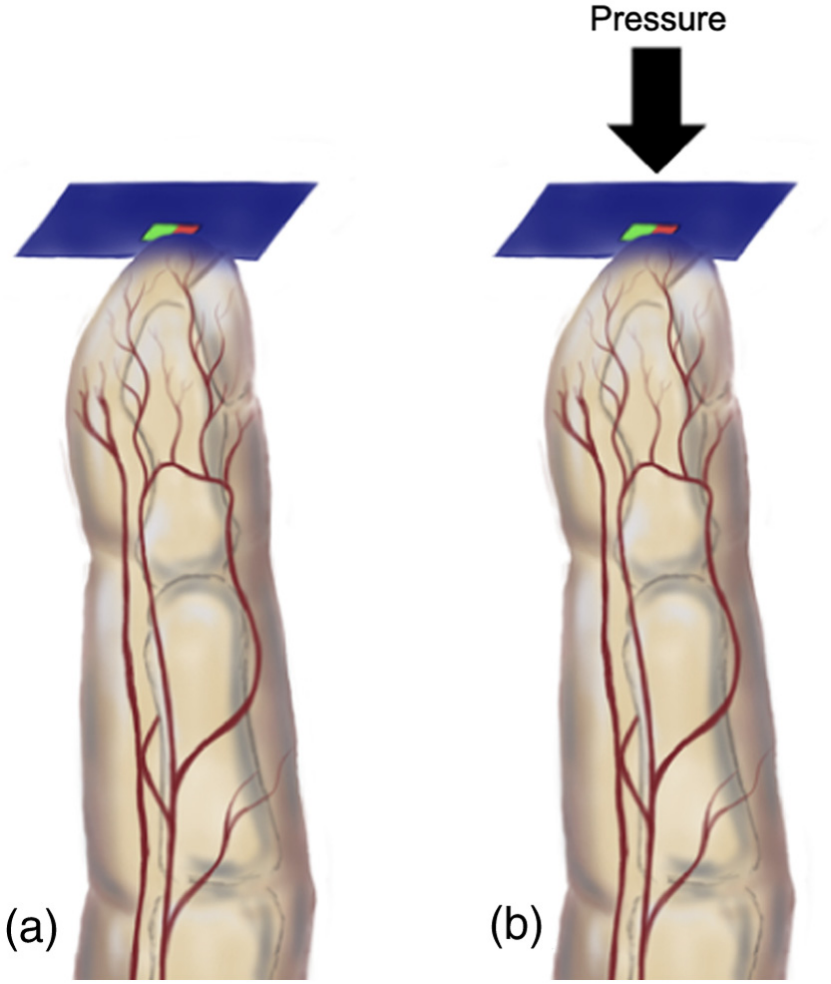
Signal acquisition in experiment 1. (a) Signal acquisition without pressure and (b) signal acquisition with pressure.

The aim of this first experiment was to determine whether green PPG (GPPG) and green PPG first derivative (GPPGFD) could be utilized effectively for the detection of changes in peripheral microcirculation. First, the signal was acquired continuously for 90 s at resting state. Then, the same experimenter applied moderate pressure to the PPG sensor against the fingertip for all participants, ensuring minimal variability in the applied pressure, as shown in Fig. 1. This pressure was sustained for an additional 90 s, while signals were acquired. The aim was to quantitatively demonstrate whether there was an observable change in the GPPG and GPPGFD when pressure was exerted on the sensor. This change could be partially attributed to the potential occlusion of the superficial capillaries due to its anatomical distribution which would confirm that the obtained signal corresponded to microcirculation, avoiding the need for repeating the compression test in the following experiment in the sublingual area, because of the limitations and discomfort that could be caused in the oral cavity.

The second experiment aimed to assess the sublingual microcirculation through 15 features calculated from GPPG and GPPGFD acquired using the same PPG sensor.

**Fig. 2:**
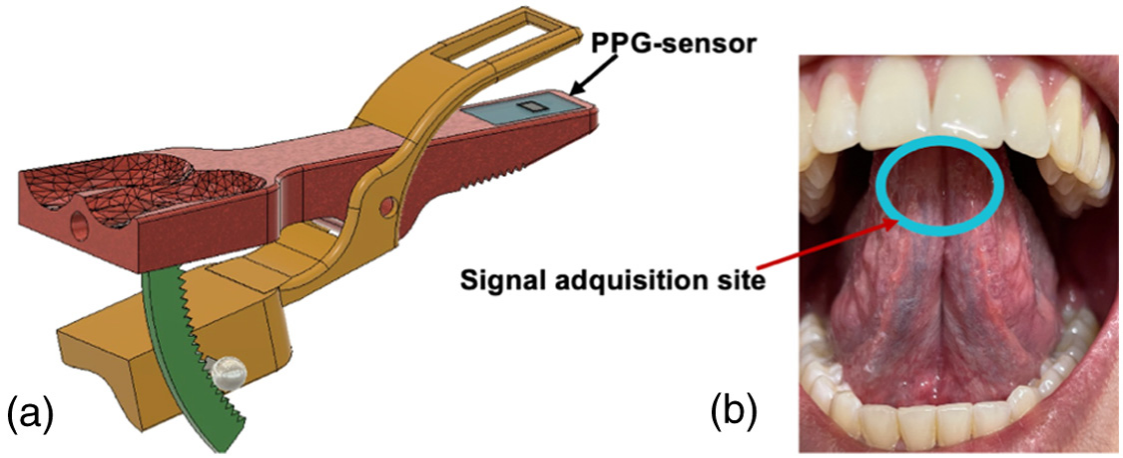
Probe and signal acquisition site during experiment 2. (a) Probe for immobilizing the tongue and acquiring PPG signals from the ventral surface. (b) Ventral surface of the tongue where the PPG sensor was positioned for signal acquisition.

A probe, shown in Fig. 2, was designed and developed to serve as a reliable and efficient tool for immobilizing the tongue and acquiring PPG signals during the experiment protocol, while maintaining the microcirculatory blood flow and minimizing any potential movement that could compromise data accuracy. Its ergonomic design facilitates easy handling through a pinch grip, utilizing the thumb, second and third fingers. By firmly grasping the tongue, the probe ensures its stability throughout the measurement process. In addition, the probe allows direct contact between the LEDs and the tongue’s ventral surface. To maintain hygiene standards, the sensor was covered with a 6-gauge film stretch made of linear low-density polyethylene, which was replaced after each use.

As with the first experiment, data acquisition took place in a dark room to avoid ambient light influence. Signals were acquired continuously for 2 min from all participants.

In both experiments, signals were acquired using a custom-made LabVIEW virtual instrument.

## 3 Signal Processing

Signals were processed offline using MATLAB R2023a.

### 3.1 Experiment 1

Each acquired signal was manually and individually evaluated by the team members. The evaluation methodology, inspired by the proposal of Tang et al., classified PPG signals as excellent when both the systolic and diastolic waves were identifiable, acceptable if the systolic or diastolic waves were not identifiable, but the heart rate could still be determined, and unfit when neither the heart rate could be determined nor the systolic and diastolic waves could be distinguished.^38^

The processing of the data included the rescaling of the voltage measurement to span from 0 to 3.3 V, band-pass filtering (cut-off frequencies 0.025 and 7 Hz), zero-padding, and the calculation of the first derivative from the PPG. The first derivative of the PPG describes the rate of change of volume in time. This variable was of interest since the microcirculatory flow features a noteworthy decline in velocity compared to the arterial flow.^4^ Hence, the changes in microcirculatory flow are expected to affect the rate of change of the volume, i.e., the first derivative of the PPG. From a frequency-domain point of view, it was expected that the first derivative of the PPG should tend to lower frequencies in the microcirculatory flow, due to the transition from an average arterial velocity of 500 to 0.5  mm/s within the smallest vessels, such as capillaries.^4^

Then, PPG signals and their derivatives were analyzed in both time and frequency domains, from which 15 features to evaluate the GPPG and GPPGFD were obtained. The extracted features are described in Table 3.

**Table 3:** Features for microcirculatory blood flow assessment.

Assessed features	Abbreviation	Domain
Green flow maximum amplitude (average of the signal peaks)	GFMAX_A	Time
Green flow minimum amplitude (average of the signal valleys)	GFMIN_A	Time
Green flow RMS value	GFRMS	Time
Adjusted green flow RMS value (between 0.025 and 0.21 Hz)	ADJ_GFRMS	Time
Perfusion index from green PPG	PI	Time
Major peak of the green flow spectrum. (Peak value of the frequency component with the highest amplitude in the spectrum of green flow)	MAJ_PGFS	Frequency
Minor peak of the green flow spectrum (maximum peak of the green flow spectrum between the filter’s low cut-off frequency and 0.25 Hz)	MIN_PGFS	Frequency
Ratio between the minor–major peak of the green flow spectrum (ratio between the minor and major peak amplitude of the green flow spectrum)	R_MINMAJ	Frequency
X-axis centroid position of the green flow spectrum (between the filter’s low cut-off frequency and the central point between the major peak and its first harmonic)	X_CP_GFS	Frequency
Y-axis centroid position of the green flow spectrum (between the filter’s low cut-off frequency and the central point between the major peak and its first harmonic)	Y_CP_GFS	Frequency
X-axis adjusted centroid position of the green flow spectrum (between the filter’s low cut-off frequency and the major peak location in green flow minus 0.5 Hz)	X_ADJ_CP_GFS	Frequency
Y-axis adjusted centroid position of the green flow spectrum (between the filter’s low cut-off frequency and the major peak location in green flow minus 0.5 Hz)	Y_ADJ_CP_GFS	Frequency
Area under the curve of the minor peak in the green flow spectrum (between 0.025 and 0.21 Hz of green flow spectrum)	AUC_MIN_PGFS	Frequency
Area under the curve of the major peak in the green flow spectrum (within a 0.1 Hz range around the maximum peak of the green flow spectrum)	AUC_MAJ_PGFS	Frequency
Ratio between the area under the curve of the minor–major peaks (ratio of the area under the curve of the minor–major peaks in the green flow spectrum)	R_AUC_MINMAJ	Frequency

### 3.2 Experiment 2

The assessment of the signal quality and classification, as well as the preprocessing and feature extraction steps from the first experiment were repeated with the signals acquired in the second experiment.

## 4. Statistical Analysis

For the first experiment, the quantitative variables were characterized using the median values and interquartile ranges (IQR), due to the sample size and the non-normal distribution of the data. The normality of the data was evaluated through quantile–quantile plots and Kolmogorov–Smirnov test.

Paired Wilcoxon signed-rank tests were performed to compare each of the 15 extracted features at baseline and with pressure being applied to the sensor. Similarly, quantitative variables extracted from the data acquired during the second experiment were characterized using the median values and IQR, after normality of the features was again assessed through quantile–quantile plots and Kolmogorov–Smirnov test.

All statistical analyses were performed using Minitab 21.1 software.

## 5 Results

### 5.1 Experiment 1

**Fig. 3:**
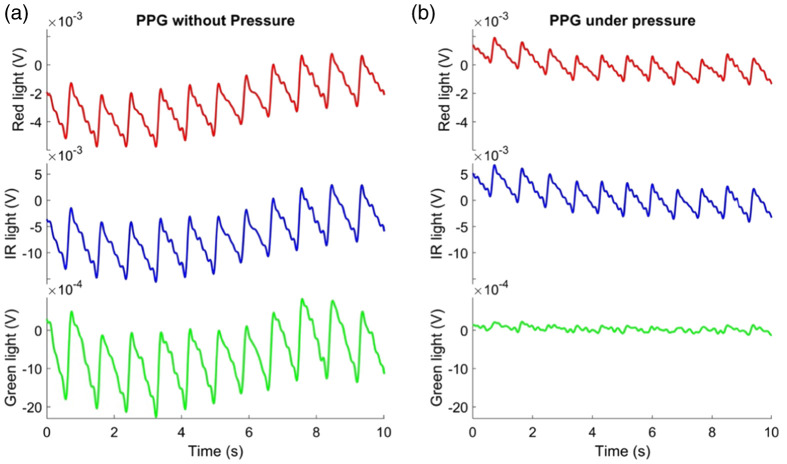
PPG signal (in relative units of volts) comparison from red, IR, and green light in the first experiment. (a) Signal in rest state condition; (b) signal in rest state condition while pressure was applied.

**Fig. 4:**
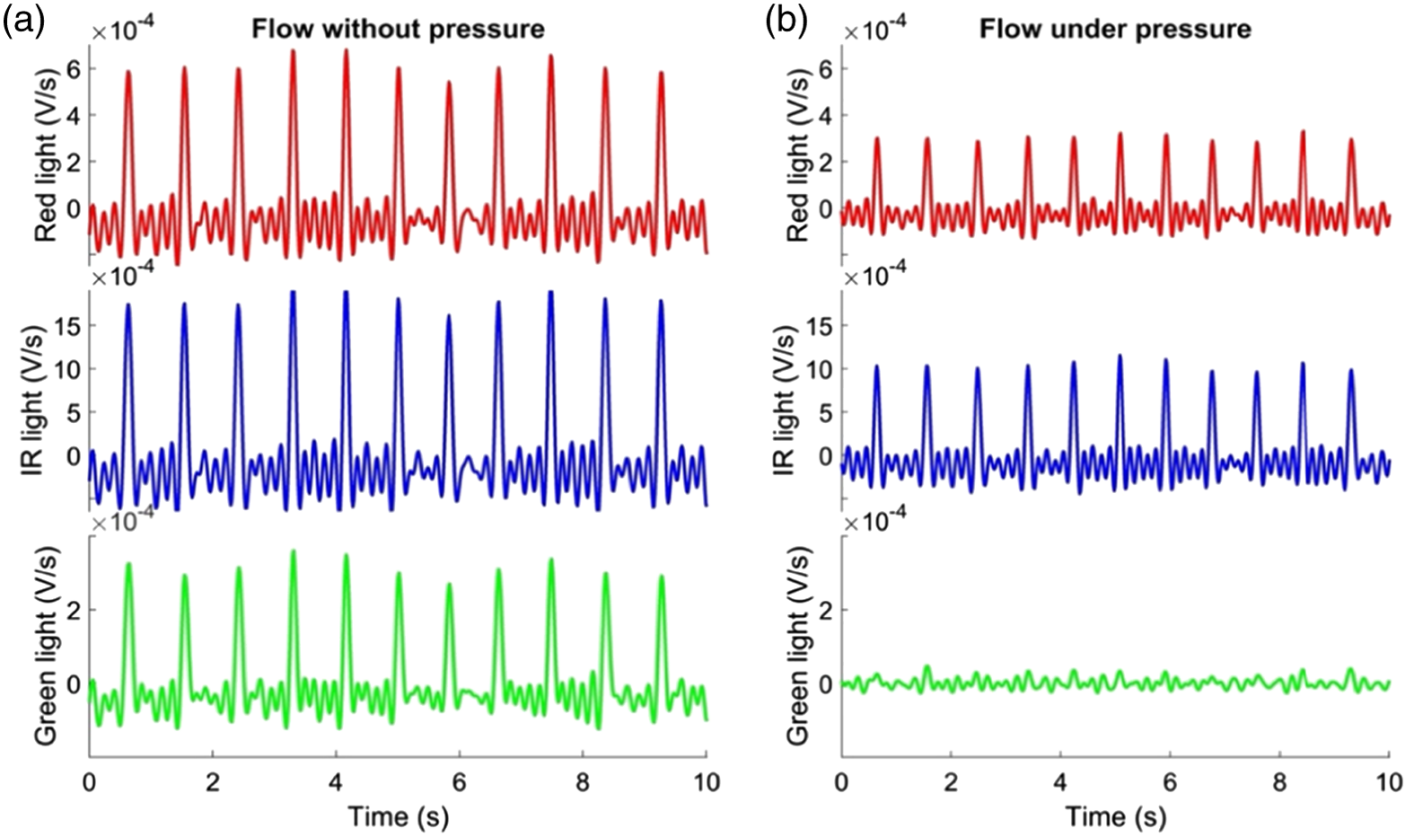
Flow derived from PPG signal (in relative units of volts) comparison from red, IR, and green light in the first experiment. (a) Flow in rest state condition and (b) flow in rest state condition while pressure was applied.

**Fig. 5:**
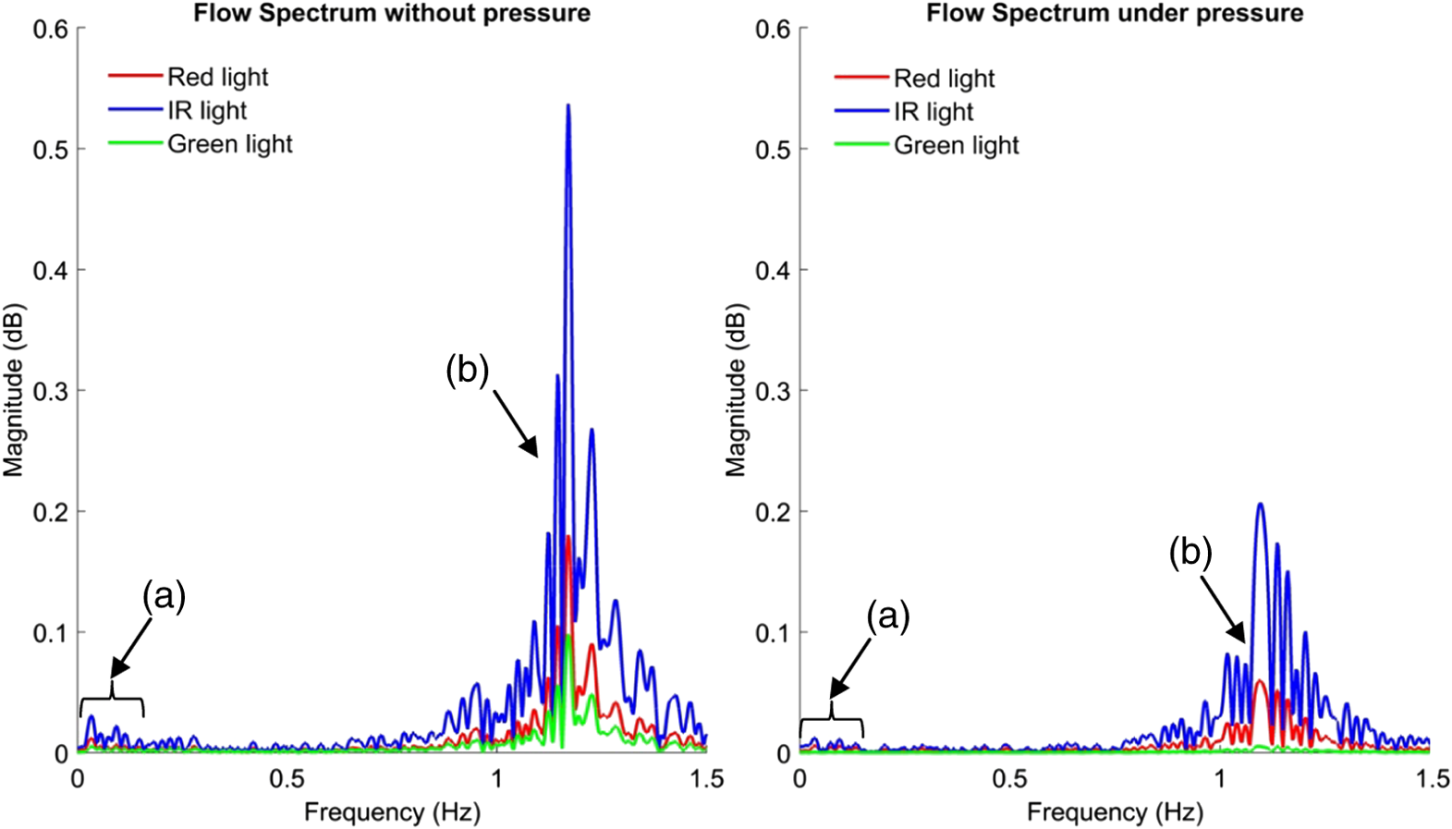
Flow derived from PPG signal spectrum comparison from red, IR, and green light in the first experiment. (a) “Low frequency” components and (b) “high frequency” components.

The PPG signals and their first derivative before and while exerting pressure on the sensor were compared. Figures 3 and 4 show these signals acquired from one of the subjects, revealing noticeable baseline wandering. This variation can be associated with the filtering cutoff frequencies of 0.025 to 7 Hz, which make the signal susceptible to interference from the respiratory rate. In order to mitigate the impact of respiration on the PPG signal, some features were extracted from two specific frequency ranges named: the “low frequency” [0.025 to 0.21] Hz and “high frequency” [0.6 to 1.8] Hz component. While significant changes in the morphology of the PPG and its first derivative were observed across all three assessed wavelengths (red, infrared, and green) as pressure was applied, i.e., capillaries vasoconstriction was simulated, the most pronounced alterations were observed specifically in the signals derived from the green light. Hence, all subsequent analyses are presented from features extracted from green PPG signals. The spectrum of the first derivative from the PPG signal was analyzed both at baseline and while applying pressure on the sensor, as shown in Fig. 5. The qualitative discrepancy observed while applying pressure to the sensor was also evident in the spectrum analysis, showing an amplitude decrease in the “high frequency” component. Notably, it appears that a similar divergence may occur in the “low frequency” component, though to a reduced degree compared to the “high frequency” component.

These preliminary findings provide support for the suitability of the green light wavelength in microcirculation assessment, which could be mainly attributed to its tissue penetration depth. Table 4 summarizes the median values of the features from the signals acquired at baseline and while pressure was applied on the sensor. A normality test was applied, and later the difference of medians was assessed using Wilcoxon signed-rank tests at a confidence level of 95%. The test results are available in a detailed manner on the Supplementary Table in the Supplementary Material.

**Table 4:** Summary of the behavior of the computed features from the signals acquired at baseline and while pressure was applied on the sensor.

	No pressure			Pressure		Difference of medians
Assessed features	n	Median	IQR	Median	IQR	p
GFMAX_A	31	0.000164	0.000248	0.000027	0.000012	<0.001*
GFMIN_A	31	−0.000079	0.000078	−0.000028	0.000005	<0.001*
GFRMS	31	0.000053	0.000066	0.000011	0.000002	<0.001*
ADJ_GFRMS	31	0.000032	0.00003	0.000003	0.000004	<0.001*
PI	31	2.017	2.046	0.2702	0.1097	<0.001*
MAJ_PGFS	31	0.04362	0.05704	0.00203	0.005293	<0.001*
MIN_PGFS	31	0.006215	0.003672	0.000515	0.000537	<0.001*
R_MINMAJ	31	0.1082	0.2188	0.2417	0.4045	0.098
X_CP_GFS	31	1.0715	0.3366	1.1108	0.282	0.008*
Y_CP_GFS	31	0.00799	0.01059	0.000302	0.000737	<0.001*
X_ADJ_CP_GFS	31	0.3231	0.1461	0.3634	0.1598	<0.001*
Y_ADJ_CP_GFS	31	0.00129	0.000907	0.00012	0.000072	<0.001*
AUC_MIN_PGFS	31	0.000468	0.000451	0.000032	0.000031	<0.001*
AUC_MAJ_PGFS	31	0.003543	0.004137	0.000146	0.000425	<0.001*
R_AUC_MINMAJ	31	0.1116	0.2073	0.1615	0.2714	0.352

Note: IQR, Interquartile range.

* Significant differences.

The calculated medians of the 15 evaluated features listed in Table 4 demonstrate a clear distinction between the two measurement periods, with and without pressure. It is noteworthy that these differences are evident in both the “high frequency” and the “low frequency” components. The Wilcoxon signed-rank test was employed to assess the statistical significance of the observed magnitude changes (Table 4), indicating that the hypothesis of equality was not rejected in only 2 out of the 15 evaluated features: R_MMINMAJ and R_AUC_MINMAJ. Consequently, this corroborates the value of using the GPPG and GPPGFD to assess the disruption in blood flow resulting from the compression of the superficial vessels. It is important to note that this compression is expected to primarily affect the superficial microcirculatory flow and not the flow in large, deeper vessels.

### 5.2 Experiment 2

**Fig. 6:**
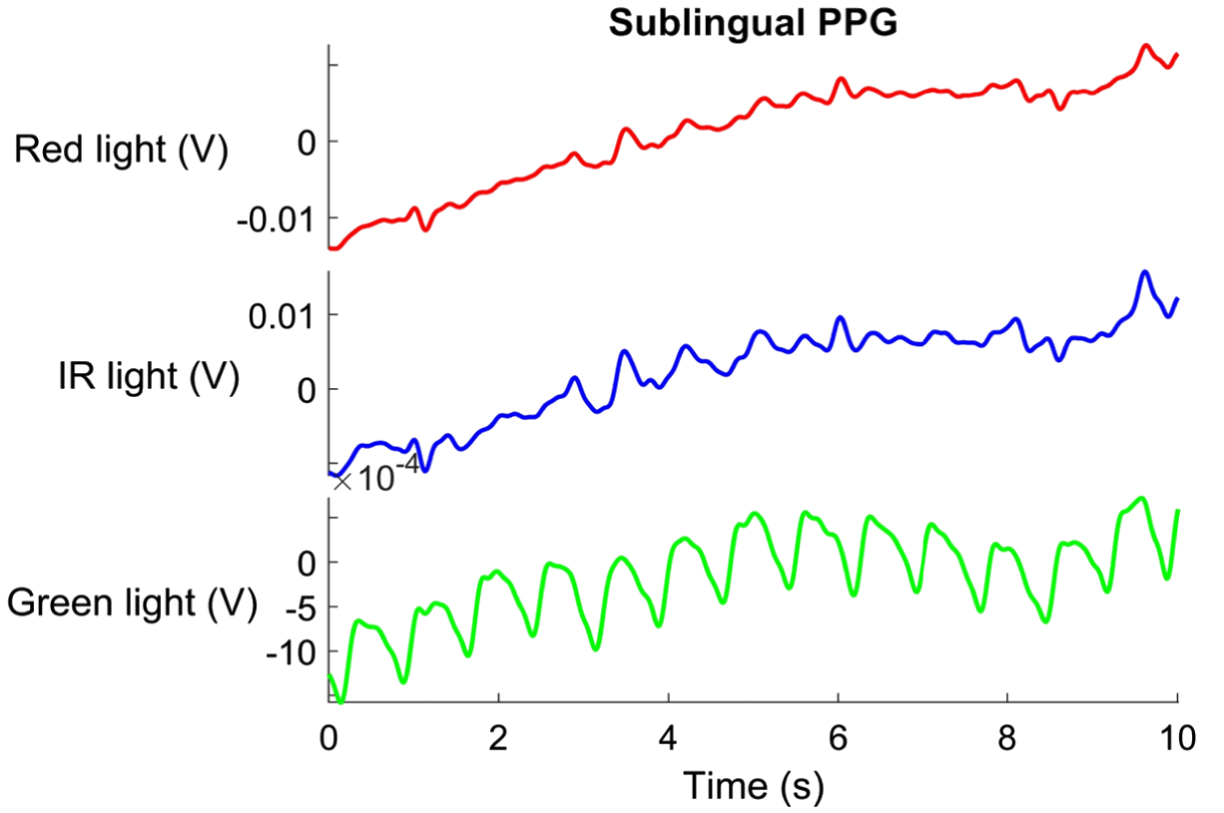
PPG signal (in relative units of volts): high-quality green light, low-quality red, and IR lights in the second experiment.

The signal’s quality classification demonstrated that only the signal from the green light met the predefined quality criteria, i.e., Fig. 6. As a result, the signals obtained from red and infrared light were excluded from further analysis. Descriptive statistics from the features extracted from these sublingual signals are presented in Table 5.

**Table 5:** Descriptive statistics of the computed characteristics in the second experiment, using PPG signals obtained from the sublingual area.

Assessed features	Unit	Median	IQR
GFAMAX_A	V/s*	0.000044	0.000049
GFAMIN_A	V/s*	−0.000041	0.000032
GFRMS	V/s*	0.000023	0,000018
ADJ_GFRMS	V/s*	0.000143	0.000247
PI	%	1.519	1.253
MAX_PGFS	dB/Hz	0.00866	0.00983
MIN_PGFS	dB/Hz	0.001793	0.002141
R_MINMAX_PGFS	—	0.2307	0.2064
X_CP_GFS	Hz	0.9766	0.2461
Y_CP_GFS	dB/Hz	0.001398	0.001578
X_ADJ_CP_GFS	Hz	0.3516	0.1217
Y_ADJ_CP_GFS	dB/Hz	0.000768	0.000767
AUC_MIN_PGFS	dB	0.00017	0.000185
AUC_MAX_PGFS	dB	0.000775	0.00097
R_AUC_MINMAX_P	—	0.2466	0.1987

Note: IQR: Interquartile range.

* Relative flow units because a calibration curve with flow values has not yet been performed.

## 6 Discussion

The microcirculation acts as a functional distribution system which regulates blood flow and thereby oxygen supply to each individual organ.^1^^,^^39^ This distribution responds to the metabolic needs of each organ under normal physiological conditions and prioritizes blood flow to vital organs during pathological situations where tissue oxygenation is compromised.^1^^,^^39^ The non-invasive monitoring of visceral perfusion through peripheral evaluation has gained attention due to the correlation between peripheral and visceral microcirculation.^1^^,^^5^^,^^9^^,^^17^ In this study, PPG has been proposed as a potential technique to evaluate sublingual microcirculation.

### 6.1 Experiment 1

PPG has been defined as an optical measurement technique that measures the volume changes in blood as it moves from the heart toward the periphery.^40^ It relies on the measurement of reflected or transmitted light when the tissue is illuminated. Therefore, the optical interaction between light and tissue plays an important role when applying this technique. This interaction is mainly affected by the optical properties of the tissue and by the wavelength of the light that illuminates the tissue. It has been shown that light with shorter wavelengths have reduced tissue penetration due to higher absorption by melanin and Hb.^31^^,^^32^^,^^34^^,^^36^ Hence, it was hypothesized that green light would offer the best performance for microcirculation assessment from peripheral tissue. Green light, which features wavelengths between 470 and 550 nm, is expected to reach the papillary dermis where the capillaries of the upper dermis are located,^31^^,^^32^^,^^34^^,^^36^ while other wavelengths, such as red and infrared, would travel deeper, failing to accurately reflect microcirculation only.

Green PPG has other noteworthy qualities, including its strong correlation with the electrocardiogram when assessing pulse frequency, and its minimal susceptibility to motion artifacts compared to other wavelengths.^33^ Considering these characteristics and the depth at which the blood vessels involved in microcirculation are located, experiment 1 aimed to validate the capacity of green light PPG to assess peripheral microcirculation. This served as a crucial basis for experiment 2, which focused on evaluating sublingual microcirculation.

In the first experiment, the aim was to understand the behavior of the red, infrared, and green PPG when finger microcirculation was obstructed. Most of the analyses done were based on the behavior of the first derivative of the obtained PPG signals. This was done due to the relationship between PPG and blood volume. Although PPG is measured in volts and indirectly reflects the volume of blood in the tissue, it could be assumed that the variation in this measure of volume in time is a reflection of blood flow (Q), as described in Eq. (1).^41^ Thus, the PPG first derivative could be considered as an indirect measure of blood flow.

Equation (1) **Flow rate derived from volume:**

$$Q=\frac{d(\mathrm{Vol}(t))}{dt}=\frac{d(\mathrm{PPG})}{dt}.$$ (1)

However, the PPG signal does not represent the behavior of one single vessel but corresponds to the behavior of multiple blood vessels present in the region illuminated. As a result, the flow, i.e., the PPG first derivative, could be considered as the blood flow across all the vessels where the PPG signal is detected.

Considering the characteristics of blood flow in the microcirculation, which exhibits a clear decrease in velocity compared to arterial flow, and the previously mentioned attributes of short-wavelength PPG, the first derivative of the green PPG (GPPGFD) was expected to enhance the accuracy of microcirculation assessment.^4^^,^^31^^,^^32^

In experiment 1, the features extracted from GPPG and GPPGFD, including the values of specific measurements related to low-frequency components (ADJ_GFRMS, MIN_PGFS, X_ADJ_CP_GFS, Y_ADJ_CP_GFS, and AUC_MIN_PGFS) showed statistically significant differences when a pressure was applied on the sensor (Table 4), and there was a clear difference in the behavior of the signals and their frequency spectra. These findings suggest that the GPPG and GPPGFD could be used to accurately evaluate peripheral microcirculation. It is important to clarify that the applied pressure was relatively low, in order to avoid affect deep blood vessels that are not part of the microcirculation.

Due to the reduced blood flow velocity in the microcirculation, promising results for microcirculation assessment were expected from the features that characterize the low-frequency band of the GPPGFD spectrum. However, a difference was not seen in the behavior of the features related to the low (ADJ_GFRMS, MIN_PGFS, X_ADJ_CP_GFS, Y_ADJ_CP_GFS, and AUC_MIN_PGFS) and the high frequency bands (MAJ_PGFS, X_CP_GFS, Y_CP_GFS, AUC_MAJ_PGFS). Further research is needed to better understand the contribution of these low-frequency components in the microcirculation.

The perfusion index (PI) has been described as a reliable indicator of changes in blood flow dynamics.^42^ The results obtained for this particular feature suggest that it could be useful in the evaluation of the microcirculation, even if measured from green PPG.

### 6.2 Experiment 2

After analyzing the results of the initial experiment and assuming that green light could efficiently penetrate the microcirculation in the tongue, with the expectation that the capillaries response in the fingertip and tongue would be similar, a decision was made to not apply pressure during the experiment 2, to avoid causing additional discomfort in the subjects, which aligns with the reason why NIRS and VOT are not suitable in this area. Experiment 2 aimed to assess the capability of measuring green PPG signals and their first derivative from the tongue, using a custom-made probe.

The same 15 features were extracted from the obtained green-based PPG signals and their first derivative. Although the compression applied when measuring the signals was not assessed in this first pilot study, it was shown that the features could be extracted from the sublingual signals, which are expected to reflect the state of sublingual microcirculation, mainly due to the depth of the capillaries in this tissue and the reach of the green light. Moreover, there is a lower presence of melanin on the ventral surface of the tongue and floor of the mouth, which in turn reduces the presence of chromophores other than Hb,^43^^,^^44^ making it a suitable area for measuring PPG with shorter wavelengths, and increasing the certainty that the obtained signal is a reflection of the interaction of light with an intravascular chromophore. Therefore, the analysis of the GPPG and GPPGFD in the sublingual region suggests that it reflects microcirculatory behavior.

Since this is the first study in which these features are extracted from sublingual PPG as a measurement of microcirculation, and due to the technological difficulties for obtaining a reference value using a gold standard, future studies with larger sample sizes are needed to relate the measured features with an actual value of microcirculatory flow. Nonetheless, the descriptive statistics from the 15 features are presented in this study as a first reference for describing sublingual microcirculation in a sample of healthy individuals.

### 6.3 Limitations

This study has some limitations. First, the sample size is relatively small and was determined based on a convenience approach. Nonetheless, due to the homogeneity of the samples, the obtained results show the potential of the technique for microcirculation assessment. Second, and particularly for the first experiment, the pressure applied to the sensor to occlude microcirculatory flow was not standardized. However, when positioning the sensor against the most distal portion of the finger, the distal phalanx acts as a barrier limiting compression and reducing the chance of light reaching deeper blood vessels. For the second experiment, involuntary tongue movements represent another limitation, and these could affect the extracted features. The design of the custom-made probe was done in order to diminish the effects of these movements on the acquired signals as much as possible, and the measurements were collected in short periods of time in an attempt to reduce the probabilities of having an involuntary movement affecting the signal. Moreover, the signals included in the analysis were manually selected based on their quality. Finally, the results obtained have not been compared with a gold standard technique for microcirculation evaluation or with features from other short wavelengths. Future studies should aim to correlate the behavior of the measured features with the gold standard, both in healthy and diseased subjects.

## 7 Conclusions

The evaluation of sublingual microcirculation using videomicroscopy has provided valuable insights into hemodynamic monitoring and tissue perfusion. However, its widespread adoption has been limited by technical complexities and associated costs. In this study, 15 features were extracted from green-light PPG signals and their first derivative, from which at least 13 effectively detected changes in peripheral microcirculation. Moreover, a proof-of-concept study was performed, showing that these features can also be measured for the characterization of sublingual microcirculation through a custom-made probe that captures PPG signals from the ventral surface of the tongue. This study offers a first approach to sublingual microcirculation monitoring that could potentially serve as a cost-effective, rapid, and user-friendly alternative to video microscopy. Further research is necessary to determine the possible origin from the signal and the correlation of these features with changes in microcirculation assessed using a gold standard, as well as to optimize the signal measurement and processing from sublingual area.

## Data Availability

The data that support the findings of this article are not publicly available due to privacy. They can be requested from the corresponding author via email.
