# Supplementary material for: Non-invasive assessment of sublingual microcirculation using flow derived from green light PPG: evaluation and reference values
Source: J Biomed Opt. 2024 Jan 5;29(1):017001. doi: 10.1117/1.JBO.29.1.017001 (PMC10768685; doi:10.1117/1.JBO.29.1.017001)
Supplement: Supplementary file 1 [file JBO_029_017001_SD001.pdf]

Kolmogórov-Smirnov test experiment 1		
**Assessed features**	**No pressure**	**Pressure**
	p	***p***
**GFMAX_A**	0,033	<0,010
**GFMIN_A**	0,034	<0,010
**GFRMS**	0,058	<0,010
**ADJ_GFRMS**	0,093	<0,010
**PI**	0,040	0,010
**MAJ_PGFS**	<0,010	<0,010
**MIN_PGFS**	<0,010	0,095
**R_MINMAJ**	<0,010	<0,010
**X_CP_GFS**	>0,150	>0,150
**Y_CP_GFS**	<0,010	<0,010
**X_ADJ_CP_GFS**	>0,150	>0,150
**Y_ADJ_CP_GFS**	<0,010	0,046
**AUC_MIN_PGFS**	>0,150	<0,010
**AUC_MAJ_PGFS**	0,043	<0,010
**R_AUC_MINMAJ**	<0,010	0,039

Wilcoxon signed-rank test experiment 1					
**Assessed features**	**Difference of medians**	**Confidence Interval**	**Wilcoxon statistic**	***p***	**Confidence level %**
**GFMAX_A**	0,0001613	(0,0000987; 0,0002275)	496	<0,001	95,1
**GFMIN_A**	-0,0000598	(-0,0000822; -0,0000392)	0	<0,001	95,1
**GFRMS**	0,0000483	(0,0000307; 0,0000683)	496	<0,001	95,1
**ADJ_GFRMS**	0,0000252	(0,0000185; 0,0000354)	496	<0,001	95,1
**PI**	1,8431	(1,28016; 2,62076)	496	<0,001	95,1
**MAJ_PGFS**	0,0483589	(0,0335609; 0,0692153)	496	<0,001	95,1
**MIN_PGFS**	0,0057096	(0,0044225; 0,0069699)	496	<0,001	95,1
**R_MINMAJ**	-0,0892599	(-0,218211; 0,0244157)	163	0,098	95,1
**X_CP_GFS**	-0,0489276	(-0,0946530; -0,0121476)	113	0,008	95,1
**Y_CP_GFS**	0,0080254	(0,0058290; 0,0116911)	496	<0,001	95,1
**X_ADJ_CP_GFS**	-0,0531066	(-0,0780156; -0,0301680)	45	<0,001	95,1
**Y_ADJ_CP_GFS**	0,0013384	(0,0010840; 0,0016637)	496	<0,001	95,1
**AUC_MIN_PGFS**	0,000465	(0,0003695; 0,0005834)	496	<0,001	95,1
**AUC_MAJ_PGFS**	0,003815	(0,0027071; 0,0050283)	496	<0,001	95,1
**R_AUC_MINMAJ**	-0,0366373	(-0,137102; 0,0498321)	200	0,352	95,1
